# Sports nutrition knowledge as a predictor of food choices in NCAA division I athletes

**DOI:** 10.1080/15502783.2025.2550165

**Published:** 2025-08-30

**Authors:** Sophie Gulleyan, Celia Lemieux, Mason Margut, Ethan Werlau, Jamie McAllister-Deitrick, Kimberly Michelle Singleton

**Affiliations:** Coastal Carolina University, Department of Kinesiology, Conway, SC, USA

**Keywords:** Sport nutrition, food choice, performance, awareness

## Abstract

**Background:**

It is well known collegiate athletes are not meeting their nutritional recommendations based on their activity level. This lack of nutritional intake can lead to poor sport performance, an increase in risk of injury, and overall poor health. Researchers have suggested that athletes are not meeting these recommendations as they have shown to have poor knowledge regarding sport nutrition, which ultimately leads to poor dietary behavior. Therefore, we assessed the sports nutrition knowledge (SNK) of Division I (DI) male athletes, as well as factors that influence their food choices.

**Methods:**

Thirteen NCAA DI male soccer players completed a demographic questionnaire, Abridged Nutrition for Sport Knowledge Questionnaire (ANSKQ), and Athlete Food Choice Questionnaire (AFCQ), with subscales of nutritional attributes of the food, emotional influence, food and health awareness, influence of others, usual eating practices, weight control, food values and beliefs, sensory appeal, and performance.

**Results:**

The overall average SNK score was 43.52 ± 14.01, which is classified as “poor.” Pearson correlation analyses revealed athletes exhibited significant, positive correlations between SNK and nutritional attributes (*r* = .60, *p* < .029), usual eating practices (*r* = .55, *p* < .05), weight control (*r* = .61, *p* < .028), and performance (*r* = .75, *p* < .003).

**Conclusions:**

The results indicate that this population overall has poor SNK. The findings suggest that as SNK increases nutritional attributes, usual eating practices, weight control, and performance tends to positively increase. This could suggest that an increase in SNK leads to better nutrition decisions. Therefore, findings support the need for implementation of a sport nutrition education intervention to increase SNK and to further investigate actual dietary intake among this population.

